# Towards building equitable health systems in Sub-Saharan Africa: lessons from case studies on operational research

**DOI:** 10.1186/1478-4505-7-26

**Published:** 2009-11-25

**Authors:** Sally Theobald, Miriam Taegtmeyer, Stephen Bertel Squire, Jo Crichton, Bertha Nhlema Simwaka, Rachael Thomson, Ireen Makwiza, Rachel Tolhurst, Tim Martineau, Imelda Bates

**Affiliations:** 1 International Health Research Group, Liverpool School of Tropical Medicine, Pembroke Place, Liverpool, L3 5QA, UK; 2 Clinical Research Group, Liverpool School of Tropical Medicine, Pembroke Place, Liverpool, L3 5QA, UK; 3 African Population and Health Research Center, PO Box 10787 -00100 GPO, Nairobi, Kenya; 4 PATH, 3039 Makishi Road Fairview Area, Lusaka, Zambia; 5 Research for Equity and Community Health (REACH Trust), PO Box 1597, Lilongwe, Malawi; 6 Disease Control Strategy Group, Liverpool School of Tropical Medicine, Pembroke Place, Liverpool, L3 5QA, UK

## Abstract

**Background:**

Published practical examples of how to bridge gaps between research, policy and practice in health systems research in Sub Saharan Africa are scarce. The aim of our study was to use a case study approach to analyse how and why different operational health research projects in Africa have contributed to health systems strengthening and promoted equity in health service provision.

**Methods:**

Using case studies we have collated and analysed practical examples of operational research projects on health in Sub-Saharan Africa which demonstrate how the links between research, policy and action can be strengthened to build effective and pro-poor health systems. To ensure rigour, we selected the case studies using pre-defined criteria, mapped their characteristics systematically using a case study development framework, and analysed the research impact process of each case study using the RAPID framework for research-policy links. This process enabled analysis of common themes, successes and weaknesses.

**Results:**

3 operational research projects met our case study criteria: HIV counselling and testing services in Kenya; provision of TB services in grocery stores in Malawi; and community diagnostics for anaemia, TB and malaria in Nigeria. **Political context and external influences: **in each case study context there was a need for new knowledge and approaches to meet policy requirements for equitable service delivery. Collaboration between researchers and key policy players began at the inception of operational research cycles. **Links**: critical in these operational research projects was the development of partnerships for capacity building to support new services or new players in service delivery. **Evidence: **evidence was used to promote policy dialogue around equity in different ways throughout the research cycle, such as in determining the topic area and in development of indicators.

**Conclusion:**

Building equitable health systems means considering equity at different stages of the research cycle. Partnerships for capacity building promotes demand, delivery and uptake of research. Links with those who use and benefit from research, such as communities, service providers and policy makers, contribute to the timeliness and relevance of the research agenda and a receptive research-policy-practice interface. Our study highlights the need to advocate for a global research culture that values and funds these multiple levels of engagement.

## Background

The Ministerial Summit of Health Research in Mexico City in 2004 recognised the need for health systems research that informs action and feeds into the development of health services that reach the poor and marginalised[1].

Better utilization of research and evidence in development policy and practice can help save lives, reduce poverty and improve quality of life [2].

The links between research, policy and action are often neglected[3] and there are surprisingly few published practical examples or systematic understanding of how to bridge the interface in sub Saharan Africa[2,4,5] Better conceptual and practical understanding that is grounded in the complexity of health systems and poverty in Sub-Saharan Africa is required.

The aim of our enquiry was to use a case study approach to analyse how and why different operational health research projects in Africa have contributed to health systems strengthening and promoted equity in health service provision. We chose case studies in order to achieve the balance between the detailed understanding of the context that is required and the generic lessons that can be learnt [6]. By extracting information about diversity and commonality[7] from case studies, researchers can attempt to explain complex phenomena[8] and determine the influence of the time, place, politics and processes of making and conducting policy[9].

**Figure 1:**
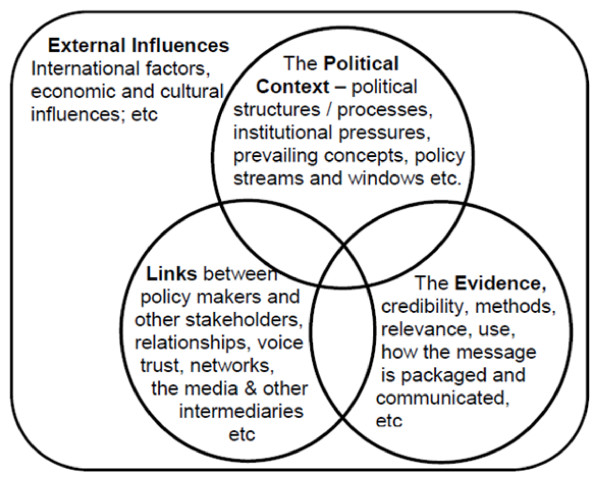
**The ODI RAPID Framework for research to policy links **[2]

The health research sector is broad, with diverse research designs and processes of impact. In this study, we focused on one specific type of research - operational research - to enable us to develop insights into the research-policy/practice interface in that area. We focused on operational research projects as these tend to be holistic in scope with the ultimate aim of understanding the operations of a complete system and improving upon it in real world settings. Operational research therefore has a strong focus on using research to improve policy, practice or service provision. First used in the military[10] operational research is growing in popularity in health systems research in resource poor contexts[11]. We frame our case study analysis within the ODI RAPID framework for analysing research to policy[2,12]. This analytical framework was developed following an extensive literature review, conceptual synthesis and testing [2]. The framework aims to enable analysis of the factors at play in the ways in which research does or does not feed into policy and practice - see figure 1.

## Methods

### Selection of case studies

The case studies were provided by the Liverpool School of Tropical Medicine's Global Health Development Group whose members have long-standing practical and multi-disciplinary experience of working in partnership in 20 countries in Sub-Saharan Africa. To minimise bias and ensure the case studies were relevant to the objectives of the analysis the case studies were selected according to pre-defined criteria. The criteria were devised by the Group to meet the study aim of learning from health research projects in African that have contributed to health systems strengthening and promoted equity. The group included principal investigators involved in the research. The selection criteria for the case studies focused on both commonality and diversity in order to be able to interrogate the issues at stake.

Criteria for commonality: Recent or current research projects that:

1. Demonstrated impact on health policy and practice, i.e. through published national guidelines, or change in policy or practice at national or district level

2. Use operational research approaches

3. Include an equity or pro-poor perspective

Criteria for diversity: recent or current research projects that:

4. Took place in different regions of Africa

5. Focused on different aspects of health systems research (e.g. TB, HIV and laboratory systems)

6. Operated at different health system tiers (e.g. national, district, community tiers)

3 case studies out of a possible 15 operational research projects on health in sub-Saharan Africa identified by the Global Health Development Working Group best met our criteria of commonality and diversity. These were:

• CS 1 Scaling up voluntary counselling and testing services in Kenya

• CS 2 Working with community providers to increase tuberculosis case finding in Malawi

• CS 3 Developing quality laboratory diagnostic systems in Nigeria

All three case studies met the first criteria of having demonstrated impact on health policy and practice at national or district level. In **Kenya (CS1) **the operational research fed into the development of national guidelines on HIV voluntary counselling and testing and a comprehensive national quality assurance plan for VCT. In **Malawi (CS2) **the findings from the community based research informed a new National TB Programme (NTP) strategy to incorporate gender sensitive and equitable engagement with informal providers within District Implementation Plans. In **Nigeria (CS3) **several aspects of the initiative have been incorporated into national policies and action plans, and at state level the close involvement and coaching of teams of laboratory supervisors means that there are advocates within each state who can champion the community diagnostics approach. In all three cases, the research was designed with these impacts on national policy as a stated aim and the links to policy makers or policy making processes were constituted from the outset. For example, in Kenya, the research team were part of the national HIV voluntary counselling and testing guidelines committee from the start of the project and were mandated by government to provide the secretariat to the national QA team. In Malawi, the operational research was carried out in careful collaboration with key partners (NTP, District Health Office, and City Assembly) who would be able to take forward the approach towards national scale-up if the results were favourable.

In all three case studies, the lessons learned from the process of operational research are also being utilised for impacts beyond the original stated aims. For example the lessons learnt from the operational research conducted in Kenya (CS1) have been replicated in other countries in the region through a twinning programme. The application of approach developed through the operational research in Malawi (CS2) is being tested by the same research team within Malawi for HIV service provision and Khartoum, Sudan for both TB and HIV service provision.

### Analysis of case studies

We developed a framework for systematically mapping the characteristics of our case studies which enabled us to identify commonality and differences in approaches and lessons for research for health systems strengthening and equity (see additional file 1). Our analysis of the research impact process was informed by the ODI RAPID framework on research to policy-links (see Figure 1) and included inputs from all authors, Principle Investigators and the Global Health Development Working Group. By incorporating these diverse perspectives the rigour of the analytical process was enhanced[13].

## Results

Through our analysis, we identified a number of common approaches to engaging with policy and practice and lessons learnt about effective approaches for enhancing the impact of operational research on health equity. These findings are presented below against the key areas identified in the ODI RAPID framework.

### External influences and policy context

We use this part of the framework to analyse the enablers/drivers behind the different operational research projects. In all cases this involved impetus from both within the research country and internationally so we have blended policy contexts and external influences.

In **Kenya (CS1) **in 2000 the Government of Kenya stated its aims to scale up quality VCT services to five sites in each of the 70 districts in Kenya [14] and incorporated this into the Kenya National AIDs Strategic Plan. At the same time new technologies in the form of rapid tests were being promoted by the WHO and donors were adding their voice and funds to the proposed scale up of services. With the government committing to support salaries of VCT sites co-located in health facilities this sustainable model became popular and up to 80% of VCT facilities to date remain in primary health care settings. It was unclear both how this model would be best rolled out, what the training and supervision requirements were and what the patterns of uptake among youth, men and women would be and whether there would be any prevention benefits[15]. Further operational research was required to elucidate some of these questions.

In **Malawi (CS2), **a partnership exists between the National TB Control Programme and an independent research organisation - Research for Equity and Community Health (REACH Trust). For a decade, the World Health Organisation (WHO) has reported that the NTP is detecting fewer than 50% of the estimated 48,000 (cases of TB arising in the country each year)[16]. Research conducted by REACH Trust (formerly EQUI-TB Knowledge Programme) highlighted the multiple challenges poor women and men were facing in accessing a TB diagnosis. These included the time and expenditure involved in multiple visits to different care providers, including multiple visits to local grocery stores[17]. These findings together with a need to try to meet the WHO target for case finding of 70%, meant that the NTP had an enthusiasm for piloting new approaches to increase tuberculosis case finding among poor and vulnerable groups. REACH Trust, together with LSTM and the NTP secured funding from the Norwegian Heart and Patient Lung Association to test whether empowering grocery storekeepers to refer of TB suspects for formal diagnosis could increase TB case finding. Grocery storekeepers in two poor, peri-urban sub-districts in the capital Lilongwe were trained. The TB case detection rate from the two intervention sub-districts more than doubled while the TB case detection rate remained static in a third, comparable sub-district (control) where there was no interaction with grocery storekeepers[18].

In Nigeria (**CS3**), the Federal and State Health Ministries recognised that in order to improve their health indicators such as maternal and child mortality rates they needed to provide better access to diagnostic services in the community. In this case there was a clear policy requirement for action, which also meant finding ways to provide better quality laboratory systems that could be incorporated into national control programmes. The DFID funded Partnership for Transforming Health Systems (PATH) which focused on 5 states responded to requests from state level to improve access to quality tests for hard-to-reach communities. By empowering and building the capacity of teams of laboratory supervisors the project set up simple diagnostic tests in 92 primary health facilities serving a population of >1,000,000. Aspects of this project are now being incorporated into national disease control programmes.

In the three case studies there was a policy momentum for action; new knowledge and new approaches were needed to fulfil policy requirements. All cases also included partnership between researchers/research organisations and policy makers and service providers from the inception of the operational research.

### Links

All three case studies included partnerships with key policy makers throughout the cycle of research beginning with problem formation (CS1, MoH, National AIDS and STD Control Programme; CS2 National TB Control Programme and CS 3 the federal and state ministries. But the links do not end here - developing partnerships at **multiple levels and with multiple players **in the health system was key in all three case studies. For example, the process of producing guidelines for scaling up HIV testing and counselling in Kenya (CS1) involved: 1) establishing a national taskforce; 2) involving counsellors from the districts in iterations and testing of the guidelines; and 3) incorporating clients' concerns into guidelines. CS2 on TB services in Malawi involved engaging with grocery store keepers, community leaders, urban assemblies, district health officers and district TB programme officers thereby forming a bridge between informal health providers and the formal health system. CS3 required effective communications between state authorities and those responsible for local government activities, as well as ensuring engagement with national policy makers and decision-makers. Harmonisation of the project activities with those of various NGOs and vertical programmes was essential to avoid duplication and for sharing of resources.

Also common across the 3 case studies and summarised in additional file 1 were **capacity building activities to consolidate links and partnership**. These were part and parcel of the operational research approach which required capacity building in the provision of new services (e.g. diagnostic tests at community facilities in CS3, and VCT within different sites and modalities in CS1) or new players in service provision (e.g. grocery store owners in CS2). In Kenya, CS1, district staff were selected as VCT support supervisors and eventually trained as trainers, able to establish sites from scratch in neighbouring districts. They were also able to offer support supervision and regular refresher courses to counsellors. The Malawi case study (CS2) involved ensuring grocery store owners could provide a screening service for TB diagnosis, training community groups in TB awareness and working collaboratively with the health workers and the NTP to develop case finding activities aimed at poor and marginalised groups. Establishing diagnostic systems in Nigeria (CS 3) involved equipping health workers with the skills to perform accurate tests as well as establishing sustainable systems between state referral and community facilities to check and improve quality. This strengthened teaching and supervision systems between secondary and primary tiers and facilitated constructive engagement with Federal policy and programme planners.

The time and resource costs of developing these multiple links and partnerships were clearly highlighted in discussion with PIs and research partners. Researchers stressed the importance of being flexible and responsive to new opportunities for partnership given the fluid and changing context - locally, nationally and globally. In Nigeria (CS3), negotiations and consensus building for joined up service delivery in different states and across different tiers within states and managing potential conflicts with the objectives of NGOs and vertical programmes, was hugely time consuming. Researchers faced challenges in embedding capacity building within different institutional cultures, especially in contexts of high staff turnover resulting in limited institutional memory. The importance and difficulty of capacity building at multiple levels - for example for research, for delivery and scale-up - was also highlighted. Difficulties were exacerbated because of limited budgets, relatively short research project time scales and the number of players involved. Further difficulties may be faced in the uptake of operational research findings and approaches in policy. For example in Malawi (CS2) the research team were concerned that the NTP had underestimated the resources that would be required to operationalise and scale up the new strategy of engaging with informal providers. The complexity of the partnerships necessary for scaling up proven approaches can also delay uptake.

In Nigeria (CS3) the long term plan is to have a dedicated unit at federal level to take forward the integration of community testing and quality assurance processes into state and national plans, but this is a complex process requiring agreement across several programmes and departments as well as involvement of external funders and NGOs.

### Evidence

We use this part of the ODI framework to analyse key issues in the creation of evidence for equity and pro-poor approaches given our explicit aim of learning from operational research that promoted policy uptake for equitable interventions. Of note was the **framing **of the research problem: for example CS3 specifically chose to focus on anaemia, malaria and TB, which typically affect poor girls, boys, women and men[19,20]and where there had been less investment in diagnostic resources (as compared to HIV for example). The aim of CS2 was to increase case finding (which is a key concern of TB programmes) amongst poor and vulnerable communities and individuals by bringing services closer to poor communities and reducing the costs and opportunity costs of TB care seeking.

The approach to **sampling **was also guided by equity considerations for example the operational research on diagnostics in Nigeria (CS 3) specifically selected hard-to reach sites on the basis of lack of access to diagnostic services, and the operational research in Malawi specifically selected poor areas in Lilongwe to pilot new approaches to case finding. CS1 included work in underserved rural health facilities and dispensaries, normalising HIV testing and linking quality assurance of services to the local community. All three cases demonstrated that new approaches to service delivery can work in poor, under-resourced areas making advocacy to scale-up successful interventions more compelling.

All the research projects also used **multiple methods **to gather robust evidence on poverty and equity. Multiple methods used included analysis of routine data from health records, exit surveys, questionnaires and qualitative and participatory methods, such as participatory gender sensitive poverty assessments. These enabled the interpretation of client and/or different community members' experiences (disaggregated for example by age, gender and socio-economic status) against a background of statistical analysis of Health Information Systems Data when investigating uptake of HIV Counselling and Testing and TB case finding (CS 1 and 2).

Developing **indicators **and assessing progress against them is a critical component of operational research[21]. The use of indicators that addressed equity considerations was central to all case studies. For example, the operational research with grocery store owners in Malawi (CS 2) used a poverty scale[22] to determine who benefited. CS1 included indicators on the number of individuals tested and receiving their results disaggregated by gender, age and HIV status[23] and also systems to incorporate feedback from counsellors. Operational research also requires ongoing assessment of progress against indicators and being responsive to emerging challenges. In Kenya, counsellor feedback on increasing numbers of clients reporting gender based violence led Liverpool VCT, Treatment and Care to collaborate with the Ministry of Health and other stakeholders to design, pilot and roll out comprehensive post rape care services within the HIV testing and counselling setting (CS 1)[24]. Limited access to HIV testing in rural areas led to the development of a mobile and outreach programme that brought services closer to potential users and made information available in sign language, through the use of deaf staff [14,25].

Researchers faced challenges in data availability on poverty and equity. Even where a poverty scale exists (CS2) it did not capture all axes of poverty and inequity, such as disability. Researchers stressed the importance of developing additional data generating strategies, to enable a more comprehensive equity analysis. Another challenge to ongoing equity analysis lies in funding the recommendations emerging from this, such as the need for mobile VCT programmes, and the development of post rape care services and VCT services for the deaf,, which arose from equity analysis of VCT scale-up data in Kenya (CS 1).

## Discussion

In this paper we use ODI RAPID's framework to frame case study lessons on how and why operational research projects focusing on different aspects of health in different countries in Sub-Saharan Africa were successful in impacting on policy and/or practice. The RAPID framework was useful in supporting our analysis and exploring the multiple overlaps between the different analytical categories. Our approach was systematic and we limited bias through a development of criteria for case study selection and a systematic analytical process involving PIs and researchers. Our cases resonate with what is already known but add critical aspects since we focused on one sector - the health sector in Sub-Saharan Africa, and analyse specific lessons from operational research.

### Developing and sustaining links

In broader debates on policy processes the traditional model of policy making as a linear process in which rational decisions are taken by those with authority and responsibility is being increasingly critiqued[26]. Policy making takes place at multiple levels (the international to local) and involves multiple players[27], relationships, and 'reservoirs of knowledge'[27]. These partnerships and networks can intensify the authoritative credibility of research[28]. Deliberate strategies to engage with a broader set of stakeholders in the community of research, policy and practice, who were not confined to policy makers, was central to the case studies. Regardless of focus or scale, researchers need to be able to forge partnerships, up and down the complex hierarchy of stakeholders involved in health policy, practice and research, and try to build credibility in the process. The case studies illustrate the importance of engaging service providers, civil society and communities in addition to policy makers and bureaucrats. Service providers have been particularly neglected, but play a critical role in interpreting and implementing policy. Working creatively to build bridges across these diverse communities is time consuming and challenging yet central to building accountability in defining research agendas, undertaking research and informing action[29,12].

### Capacity building is key

Horton and Pang argue that the 'sustainable way to improve health outcomes is to build local research and innovation capacity so that developing countries can continually improve the effectiveness, equity and efficiency of their own health systems'. Unfortunately, the literature describing practical examples of building health research capacity is scarce and tends to emphasise microlevel activities, without considering how these activities can be integrated into the wider research system or process[30]. The capacity building activities in the case studies reflect the 3 pillars of capacity building identified in a review of capacity building in resource poor contexts[30]: (1) start small, (2) Build on what exists, and (3) Develop and sustain genuine partnerships. The third principle has emerged the most clearly from our case study analysis; working in partnership with all relevant stakeholders to refine and develop research agendas and undertake research builds an appreciation of research that intensifies the effectiveness of the research to policy and practice interface[31,32].

### Evidence for equity

The need to build more equitable and gender sensitive health systems in Africa and globally has long been recognised. The role of research, and operational research in this process is less clear, and there is limited discussion on strategies that mainstream a pro-poor and gender sensitive approach throughout the research cycle. The case studies support several key themes emerging from the literature on pro-poor approaches, HIV and gender mainstreaming[33-36]. First is the need to consult across a broad range of stakeholders and develop partnerships around gender and equity in the process of constructing research agendas. Opportunities to listen to 'less heard' community and service provider views should be sought. Second is the importance of study site selection and sampling frames. For operational research, with the ultimate aim of scaling up approaches, including some of the more challenging and less resourced geographical regions is important. Third is the actual conduct of research; that is the deployment of a range of different research methods (whether clinical, quantitative, qualitative or participatory) for equity analysis, and exploring difference by socio-economic status, gender, age, (dis)ability or geography. Fourth is the development of pro-poor indicators. Indicators are already commonplace features of many health systems and health systems research, but they rarely have disaggregation relevant to the most important inequities in the research context[37]. The use of pragmatic pro-poor and gender sensitive indicators, is strategic as this powerful incentive for focusing action can help resist the 'evaporation' of attention to poverty or gender concerns, identified in the gender mainstreaming literature[33]. Making the indicators practical also enhances buy-in[38], and in turn increases the options for advocating for scale-up of promising research findings[38].

## Conclusion

Health systems are complex and fluid entities working at multiple levels; there are no simple solutions[39]. For the development of equitable and effective health systems, researchers need to embrace two inter-linked challenges. Firstly, in a context where the links between poverty, marginalisation and (ill) health are so compelling, there is an urgent need to mainstream an equity or pro-poor approach throughout the research cycle. In operational research, pro-poor indicators are essential to ensure that equity considerations do not evaporate, but are central to analysis, dissemination and scale-up. Secondly researchers need to build partnerships on many fronts: multi-disciplinary partnerships to ensure that their research does justice to the holistic and complex nature of health systems; partnerships for capacity building to promote demand, delivery and uptake of research; and partnerships with the broader research, policy and practice constituency, from communities to service providers to policy makers, to ensure the timeliness and relevance of the research agenda and a receptive research-policy-practice interface. There is no magic formula for these partnerships, as they will need to reflect different, often fast-moving, institutional contexts, the interplay between vertical and horizontal approaches to health in specific countries, and particular research foci. The levels of engagement demanded by these partnerships will take time, energy, skills and resources. "Methods for partnership development" is a new component for the evolving health systems research paradigm. We need a global research culture that values and funds these new levels of engagement from multiple sources including governments, foundations, and charities.

## Competing interests

The authors declare that they have no competing interests.

## Authors' contributions

All authors fed into the development of the criteria against which to select case studies. ST, MT, JC and IB led the design of overall approach and the analysis framework. All authors participated in the analysis and critical interrogation of key themes. All authors read and approved the final manuscript.

## Supplementary Material

Additional file 1 **Table S1: The Case studies**. This table summarises the details of the selected case studies Click here for file
