# Supplementary material for: Towards building equitable health systems in Sub-Saharan Africa: lessons from case studies on operational research
Source: Health Res Policy Syst. 2009 Nov 25;7:26. doi: 10.1186/1478-4505-7-26 (PMC2789709; doi:10.1186/1478-4505-7-26)
Supplement: Additional file 1 — Table S1: The Case studies. This table summarises the details of the selected case studies [file 1478-4505-7-26-S1.doc]

**Table S1, Additional File 1: The Case studies**

| **Case study, focus and country context** | **Organisations taking forward the operational research** | **Aim of the operational research** | **Level of activity** | **Examples of pro-poor/equity approach** | **Capacity building activities** | **M&E** | **Impact on policy/scale up** |
| --- | --- | --- | --- | --- | --- | --- | --- |
| **CS 1Scaling up voluntary counselling and testing services in Kenya** | Liverpool VCT and Care (an independent Kenyan NGO and service provider) in collaboration with LSTM and with funding from DFID, USAID and CDC | Support the Government of Kenya to scale up quality voluntary HIV counselling and testing services (VCT) | Practical support given at each level:  **National**: taskforce established, guidelines and training manual developed. Site registration system and comprehensive QA system established.  **District Level:** Regular meetings; joint visits for site selection, district supervisors trained and counsellors selected.  **Site level:** HIV counsellors trained and support supervision instigated | Sites selected on basis of coverage and HIV prevalence.  Regular data analysis of access to VCT services on the basis of age, gender and rural/urban location | National taskforce members involved in comprehensive quality assurance systems.  District staff selected as VCT support supervisors and eventually trained as trainers, able to establish sites from scratch in neighbouring districts.  Site counsellors offered support supervision and regular refresher trainings | Number of individuals tested and received their results disaggregated by gender, age and HIV status  Number of districts with at least one site per 100,000 of the population aged 15-45  Number of sites with a quality assurance system in place for HIV counselling and testing services | Provided evidence base for national guidelines on VCT published in 2001  Provided pilot for a comprehensive national QA system. |
| **CS 2:Developing links with community providers to increase tuberculosis case finding in Malawi** | Extending Services to Communities Project, LSTM, REACH Trust and Malawi NTP, with funding from Norwegian Heart and Lung Patient Association and DFID-funded EQUI-TB Knowledge Programme | Utilising the informal sector (grocery storekeepers) in TB recognition and referral to increase access to quality TB services and promote TB case detection. | **National Level:** Engagement of NTP policy makers to ensure strong platform for extending the TB services  **District Level:** Regular meetings between district health authorities and community groups to ensure support for initiative  **Site Level:** Grocery storekeepers trained in symptom recognition and referral mechanisms | Grocery stores selected as they are currently a key and frequent point of access for the poor. ^[17]^  Sites selected as poor sub-districts of the city, by both qualitative and quantitative measures. | Storekeepers’ capacity strengthened through regular training and follow up. Community groups trained in TB awareness and health promotion activities.  Local researchers trained in implementing multidisciplinary intervention research. | Increase in early care seeking for chronic cough.  Significant increase in proportion of chronic cough and smear positive cases notified at health facilities from the intervention areas.  Poverty scale used to determine who benefited.  Determination of the perceptions and acceptability of the intervention among key stakeholders. | The NTP is using the findings of this community based initiative to advocate incorporating equivalent gender sensitive and equitable engagement with informal providers within District Implementation Plans.  Approach now being exported and tested in collaboration with NTP in Sudan |
| **CS 3: Developing quality laboratory diagnostic systems in Nigeria** | Integrated into national programme activities. Supported by government funds and several external donor projects | Establish simple , quality-assured tests for anaemia, malaria and TB in hard-to-reach communities in five Nigerian states | **Federal**  Engagement of Federal policy and programme planners  **District**  Strengthened teaching/supervision systems between secondary and primary tiers | Sites selected on basis of lack of access to diagnostic services  Diagnostic services for maternal and child health prioritised | Technical and generic health workers equipped with skills to perform accurate tests  Sustainable systems established between state referral and community facilities to check and improve quality | Competence of individuals to perform tests to acceptable standards  Measurable improvements in test quality  Increase in diagnostic test coverage for hard-to-reach populations | Provided practical mechanism to implement national laboratory policy at community level and platform on which to build better access to additional diagnostic services  Generated national cohort of confident and articulate laboratory experts as Federal advisors |
